# Environmental Impact of Textile Reinforced Concrete Facades Compared to Conventional Solutions—LCA Case Study

**DOI:** 10.3390/ma12193194

**Published:** 2019-09-29

**Authors:** Lenka Laiblová, Jan Pešta, Anuj Kumar, Petr Hájek, Ctislav Fiala, Tomáš Vlach, Vladimír Kočí

**Affiliations:** 1 Faculty of Civil Engineering, CTU Prague, Thákurova 7, 166 29 Prague, Czech Republic; petr.hajek@fsv.cvut.cz (P.H.); ctislav.fiala@fsv.cvut.cz (C.F.); tomas.vlach@cvut.cz (T.V.); 2 University Centre for Energy Efficient Buildings, CTU Prague, Třinecká 1024, 273 43 Buštěhrad, Czech Republic; jan.pesta@cvut.cz; 3 Department of Environmental Chemistry, Faculty of Environmental Technology, University of Chemistry and Technology Prague, Technická 5, 160 00 Prague, Czech Republic; vlad.koci@vscht.cz; 4 Natural Resources Institute Finland (Luke), Production Systems, Tietotie 2, FI-02150 Espoo, Finland; anuj.kumar@luke.fi

**Keywords:** textile reinforced concrete (TRC), life cycle assessment (LCA), environmental impact, high performance concrete (HPC), carbon fibers, AR-glass fibers, basalt fibers

## Abstract

Pitch-faced concrete is becoming a very popular element of modern architecture in the 21st century. In particular, the demand for concrete facades is increasing globally. On the other hand, climate change, environmental degradation, and limited resources are motivations for sustainable building materials. The construction industry is one the highest emitters of CO_2_ and other greenhouse gases, in which concrete plays a major role. Thus, reduction in the volume of concrete consumption is essential to control greenhouse gases. One approach to this problem is to use textile reinforced concrete (TRC). The main aim of the present study was to compare the subtle TRC facade made of three different types of technical textile rovings (glass, carbon, and basalt) with ordinary facades reinforced by steel reinforcement (ORC). The goal was to compare the basic environmental impact potential according to product category rules (PCR) for concrete structures. The functional unit was defined as an experimental facade with an area of 60 m^2^ and a 100-year lifespan. Inventory data were elaborated for concrete, steel, and textile fiber production; the building site; service life; demolition; and final disposal. The main life cycle assessment (LCA) parameters were global warming potential (GWP), ozone depletion (ODP), acidification (AP), eutrophication (EP), abiotic depletion (ADP), and photochemical oxidant creation (POCP). All the data used in the work were related to Czech Republic. Textile reinforced concrete facades appeared to be more environmentally friendly in four of six impact categories by an average of 30%. The results of the present study revealed that, in comparison to ORC, TRC has a lower environmental impact for the given conditions and thus good potential for use in sustainable construction.

## 1. Introduction

Civil engineering is one of the largest global consumers of material resources, and producers of waste and harmful emissions. Buildings and building structures have a significant impact on the environment at a local scale as well as globally [1,2]. This sector is especially responsible for greenhouse gas (GHG) emissions [3,4] and has a significant impact on energy use [5]. Approximately 30–40% of all primary energy used globally relates to the operation of buildings [6]. 

Environmental impacts can be divided into several levels: global, regional, and local [7]. Issues at the global level concern ozone depletion, the greenhouse effect, and the related global warming. At the regional level, the most important problems are acidification of the environment and water eutrophication and at the local level consumption of local sources of raw materials, especially non-renewable resources and water, is the chief concern [7]. 

It is widely known that concrete is the second most-used material in the world after water [8,9]. According to the World Business Council for Sustainable Development report from 2009 [10], global concrete production was estimated to be approximately 25 billion t per year, which corresponds to a consumption of more than 3.8 t per person annually [4]. In the Czech Republic, as well as globally, there is a growing demand for aesthetic elements made of pitch-faced concrete. Raw concrete is not only a material for further surface treatment but is increasing in popularity in its pure form, especially for use for facade elements [11,12]. The increasing use of concrete, however, has a significant impact on the environment [13]. The use of steel reinforcement represents a significant proportion of this impact. The most significant environmental impact, mainly due to CO_2_ emissions, is cement production [14]. The global production of cement is responsible for 7% of all CO_2_ emissions [15], which has a significant effect on global warming and climate change. Worldwide cement production increased more than 12 times in the past 50 years [16] and further growth can be expected [14]. The European cement industry has subsequently undertaken a review, resulting in the identification of the best practices in the use of materials and energy and in the reduction of greenhouse gas (GHG) emissions over time, to pinpoint trends in outcomes and performance improvements [17]. Construction and its products are responsible for 30% of total CO_2_ emissions in the EU [7]. Optimization of concrete consumption and efforts to use green concrete have, therefore, become one of the most discussed topics in recent years [18,19].

There are several approaches to solve the above-mentioned problems. Clearly, one possibility is to replace concrete with completely different materials, but this is not possible in many cases due to the indisputable advantages of reinforced concrete. Partial substitution of some environmentally demanding concrete components appears to be an interesting solution [20,21], as well as the use of recycled concrete waste [22,23]. Another option is to use high-performance materials, or suitably optimized cross-sections of individual elements [24,25], or to replace steel reinforcement by non-convention reinforcement [26,27]. Textile reinforced concrete (TRC) [28] can contribute to a solution by providing two advantages: steel replacement and considerable concrete savings [29]. Such an approach is particularly suitable for non-bearing elements such as facades. TRC is a relatively new material, which has been studied, for example, at RWTH University in Aachen [30], at TU in Dresden [31], and in [32,33]. In addition, many numerical analyses of new TRC experimental elements and structures have been undertaken and presented [34,35]. Although high-performance concrete (HPC) or ultra-high-performance concrete (UHPC) used for TRC elements is generally more environmentally demanding than conventional concrete mainly because of the large amount of cement and fine admixtures, in the case of TRC, it is used considerably less because of the minimal coverage of textile reinforcement [36]. This significantly reduces the consumption of concrete, as well as the total amount of transported material. Transport is one of the key parameters in the whole life cycle assessment [37]. In addition, taking into account the multiple lifetimes of TRC elements compared to conventional concrete elements [38], this composite material proves to be very effective in terms of environmental impacts. 

The main aim of the present study was to compare the subtle TRC facade elements made of three different types of technical textile rovings (glass, carbon, and basalt) with ordinary facades reinforced by steel reinforcement (ORC) in terms of selected basic environmental impact potential. Production in the Czech Republic and Czech climatic conditions were considered for all variants. The analysis includes all distances for the transport of the individual raw materials and materials needed for production, as well as the energy flows for the specific production.

## 2. Materials and Methodology

### 2.1. Material Used

#### 2.1.1. Concrete

The details of mixtures of high-performance concrete (HPC) and conventional concrete of class C30/37 (OC) used for all types textile reinforced façade panels are described in Table 1.

#### 2.1.2. Reinforcement

For textile reinforced concrete elements, hand-made textiles from commercially available AR glass, basalt, and carbon rovings were used, and the technical properties of glass rovings are noted in Table 2. All these textile rovings were coated with epoxy resin supplied from Sikafloor 156 ® (Sika, Stuttgart, Germany) with 1100 kg/m^3^ density, 15 MPa tensile strength and 2 GPa modulus of elasticity. For OC reinforcement, 6-mm steel curry mesh was used.

### 2.2. Comparison Variants

**Figure 1:**
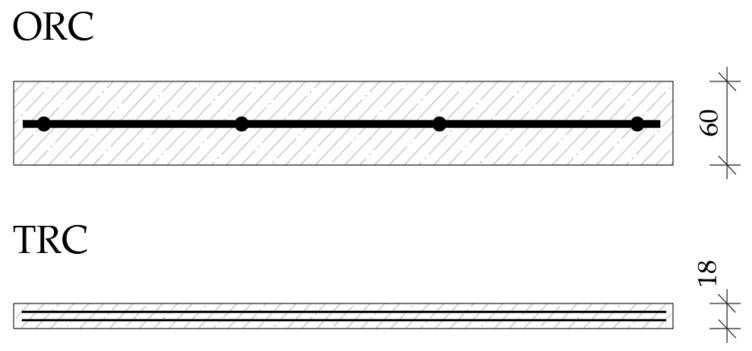
Comparison of variants (cross section of facade panels).

A total of four variants of concrete facade panels were compared:

- V1 (ORC steel): Standard concrete reinforced with a 6 mm diameter steel curry net with a mesh of 150 mm × 150 mm. Total thickness of facade boards is 60 mm (see Figure 1).
- V2 (TRC glass): High performance concrete reinforced with 2 layers of AR glass textile reinforcement. Total thickness of facade panels is 18 mm (see Figure 1).
- V3 (TRC carbon): High performance concrete reinforced with 2 layers of carbon textile reinforcement. Total thickness of facade panels is 18 mm.
- V4 (TRC basalt): High performance concrete reinforced with 2 layers of basalt textile reinforcement. Total thickness of facade panels is 18 mm.

## 3. Environmental Impacts Assessment Using Life Cycle Assessment (LCA)

Cradle-to-grave comparisons of the environmental impacts of concrete facades were carried out according to the ISO 14040:2006 standard [39], which describes the four basic assessment steps: goal and scope definition, life cycle inventory, life cycle impact assessment, and life cycle interpretation. The LCA software, GaBi Professional [40], was used to evaluate the environmental impacts of the mentioned four variants used in the present work. For concrete structures, the European standard EN 16757:2017 (Sustainability of construction works—Environmental product declarations—Product Category Rules for concrete and concrete elements) [41] was used. This standard supplements the basic rules for the product categories of construction products set out in ISO 14040:2006 for concrete and concrete elements of building and civil engineering works. Further, it defines the assessment parameters, phases, and method of impact assessment. According to product category rules (PCR) [41], the following impact categories were compared: Global warming potential (GWP), ozone depletion (ODP), acidification (AP), eutrophication (EP), abiotic depletion (ADP), and photochemical oxidant creation (POCP). All data related to the Czech Republic. Specific data for concrete production in Czech Republic were obtained from ICFconcrete 3.0 [42]. For some processes, generic data were also used.

### 3.1. Functional Unit

The concrete facade serves as a design feature, as well as a durable building envelope. It protects the building from adverse effects for as long as possible while maintaining design and mechanical parameters. The functional unit represents a measure of the function of the studied system. It provides the basis for the modelling that follows. For the comparison, an experimental facade with area of 60 m^2^ and 100-year lifespan was set as the functional unit. 

### 3.2. System Boundaries

For the comparison of facade panels, a cradle-to-grave scale was used. Therefore, all life phases of the individual variants were assessed as follows: extraction of raw materials and transport to the production plant; production of partial materials and transport to the prefabricated production plant; production, treatment, and transport to the building; installation; and use to the end of the life cycle. Some data used for modelling were obtained from cement manufacturers in the Czech Republic. However, because production is similar worldwide, these values can be considered as universally representative. The transport of individual components was calculated for production and prefabricated production plants in the Czech Republic, and these data may vary considerably for other countries. Concrete facade life cycle steps were broken down into three phases: production, use, and end of life.

#### 3.2.1. Production Phase

The production phase includes all processes from the extraction of raw materials, their transport to production plants, processing, transport to the place of production of prefabricated elements, production of prefabricated parts, treatment, storage, transport to the construction site, and their installation. For each material, the exact distance of the conveyed element from the production site to the prefabricated production plant was calculated. Subsequently, the transport of precast elements to the building site was evaluated. Transport was divided into long-distance and local. For local transport, a distance of up to 30 km was considered, and the considered vehicle was a small truck (up to 14 t total capacity, 9.3 t payload). For long-distance transport, a bigger truck was considered (40 t total capacity, 24.7 t payload). Data on concrete mixing and preparation of the prefabricated panels were set as averages of Czech concrete plants taken from ICFconcrete 3.0 [42]. Data on installation were estimated considering an amount of the materials on the construction site.

#### 3.2.2. Phase of Use

Although the lifetime of TRC panels is several times higher than that of conventional panels, it is necessary to take into account the moral lifetime, which may be decisive in the case of facade panels. For this reason, a service life of 100 years was chosen for all variants. For TRC elements, regular repairs and a possible replacement of 5% of the elements are expected during this time. In the case of conventional panels, repairs and replacement of elements in the order of 15% are expected. During the use phase, maintenance and cleaning with pressurized water was counted once every 10 years of the facade life. In addition, water for facade cleaning was estimated according to the experience of local companies.

#### 3.2.3. End of Life Cycle

In the final phase of the life cycle, work related to demolition is included, including the use of a crane and transport to a landfill. The recyclability of a particular type of reinforced concrete is not included in the assessment.

### 3.3. Life Cycle Inventory

The following tables summarize the input data used to calculate environmental impacts. Table 3 summarizes the data for the entire production process. Table 4 contains data for the phase of use, and Table 5 shows the data for the end of the life cycle.

### 3.4. Life Cycle Impact Assessment

In the environmental impact assessment phase, the individual results of the inventory analysis are linked to specific environmental impact categories, and their influence for each category is expressed with an impact category indicator. The first step in impact assessment is classification. Elementary flows from inventory results are assigned to each impact category, which can be potentially influenced by them. Then, in the next step, which is called characterization, the measure of the effect of an elementary flow on individual impact categories is calculated according to its characterization model. Such a model is a defined procedure that expresses the influence of an elementary flow on individual impact categories using a characterization factor for each flow. After classification and characterization of each flow, the result of the impact category indicator can be calculated as the summary of the results of the impact category indicators of all pollutants from the formula [43]:

(1) $$V_{\mathrm{XY}}=\;\underset{i}{\sum }\left({\mathrm{CF}}_{i,\mathrm{XY}}\;.\;\underset{r}{\sum }m_{i}\right)$$

where V_XY_ is the result of the impact category indicator XY, CF_i,XY_ is the characterization factor for substance i and impact category XY, m_i_ is the amount of elementary flow of the substance I, I represents elementary flows, and r represents emission sources.

## 4. Results and Discussion

### Life Cycle Inventory (LCI) Analysis Outputs

The LCI output data essential for LCA studies of four variants of facade panels were divided into non-renewable energy resources, non-renewable resources, and renewable resources. Table 6 shows comparison outputs of concrete facades for selected resources for their entire life cycle. The use of non-renewable energy resources varied differently for ORC and TRC. Variant V3 used a higher amount of non-renewable energy, and V4 used the least amount of energy. Variant V3 used carbon fiber as reinforcement, which consumed a high amount of lignite and natural gases. In terms of non-renewable resources consumption, V1 had almost three times as much consumption compared to textile reinforcement, and V4 used the least amount of non-renewable resources. Variant V1 consumed almost eight times as much natural aggregate in the production of ORC compared to TRC. 

**Figure 2:**
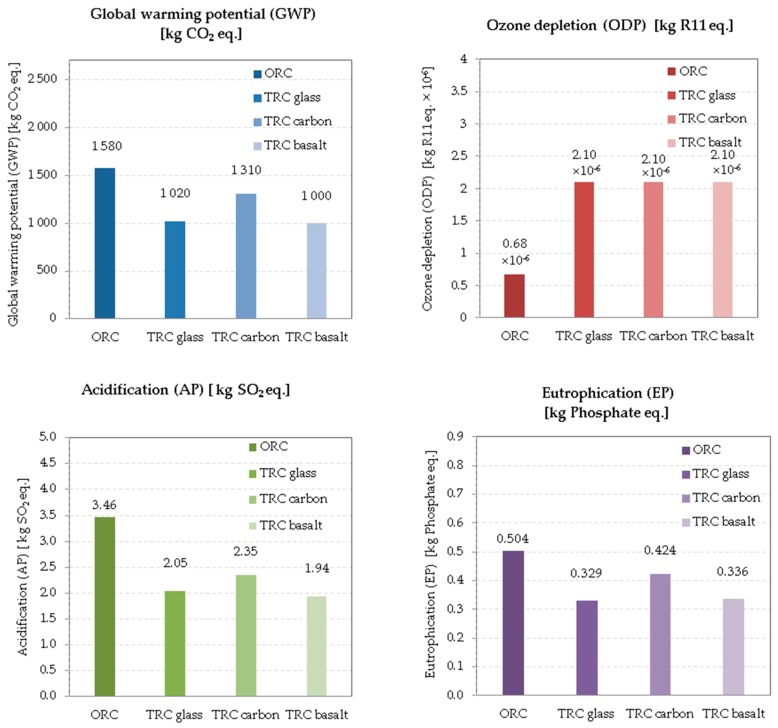

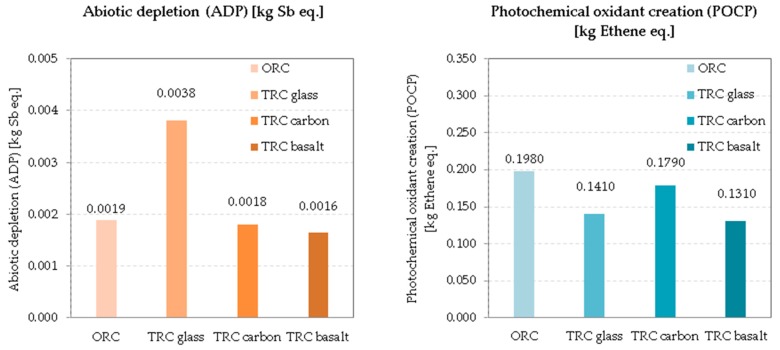
Results of individual environmental impacts.

The aggregated potential of each variant on the different environmental impacts during the all life cycle is shown in Table 7. The values are calculated according to the procedure described in Section 3.4. Comparison is evident from the graphs in Figure 2. In terms of GWP, each variant contributed differently during the entire life cycle; V1 has the highest GWP in terms of kg of CO_2_, and V4 has the lowest GWP. Similarly, AP, EP, and POCP were highest for V1 and lowest for V4 on aggregated environmental impacts at all life cycles. ADP was highest for V2 and least for V4.

**Figure 3:**
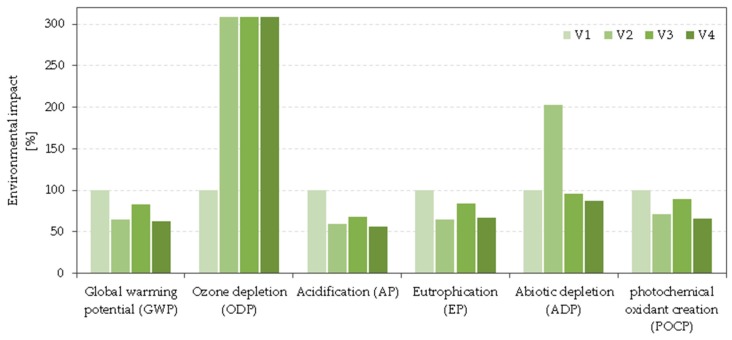
Percentage comparison between ORC and TRC on assessed environmental impacts.

Figure 3 shows the percentage comparison of environmental impacts of ORC and all three types of TRC. The GWP was 100% for V1, 50% for V2 and V4, and 75% for V3. The ADP increased to 200% for V2 compared to 100% for V1, 90% for V3 and 80% for V4. The ODP increased to 300% when ORC (V1) was replaced by TRC (V2, V3 and V4).

**Figure 4:**
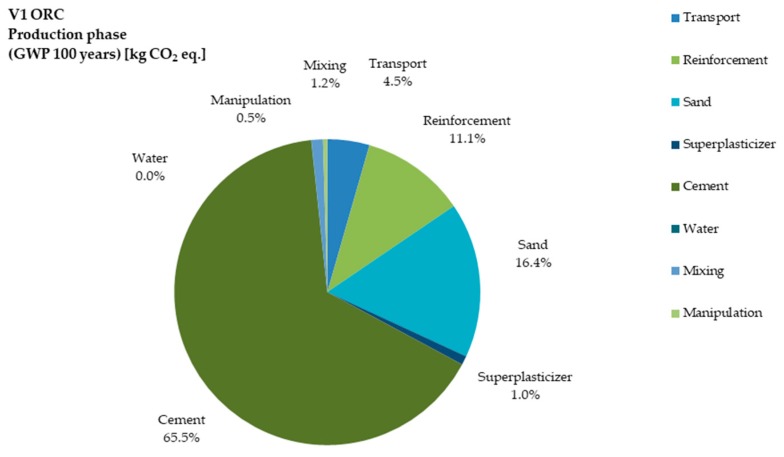
Share of impact in GWP category for the processes of V1 production.

**Figure 5:**
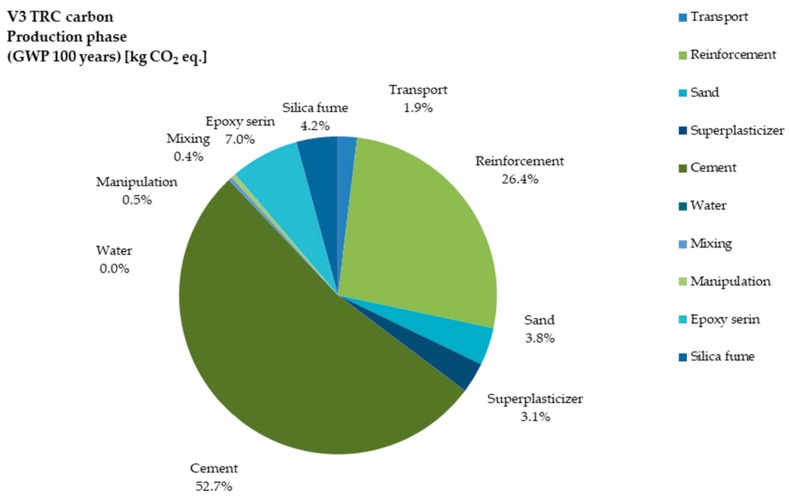
Share of impact in GWP category for the processes of V3 production.

The global warming potential (GWP) is among the most important factors for LCA of concrete development. Figure 4 and Figure 5 show the GWP of V1 and V3 during the life cycle (100 years for the present study). Cement consumption was 65.46% in V1, while it was reduced to 53.68% in V3. The potential of GWP in terms of reinforcement was 11.05% for V1 and 26.40% for V3. For V3 it increased because carbon fiber production emits more CO_2_ in comparison to steel production. In the V3 variant, epoxy resins and super-plasticizer added more GWP to TRC production, while in ORC it was negligible. Transportation contributed more GWP to ORC due to its higher weight compared to TRC. 

However, the results may vary depending on the location of the production of prefabricated elements and on the sources used, and therefore cannot be completely generalized. Nonetheless, the results show the potential for improving environmental impact by using TRC for subtle structural elements.

The calculation included detailed production data, transport data of individual elements, and partly, their service life. If we consider only the absolute life of the elements and neglect the moral life, the advantage of TRC elements would multiply, since the TRC elements have a lifespan of several hundred years. Durability is undoubtedly an advantage of this material and plays a major role in the results; however, the environmental advantage is already visible for the production phase. The indisputable advantage is, of course, in the lower weight of the final elements and in the steel replacement, which is reflected in the transport and assembly. However, it should be noted that the presented types of textile concrete have some reserves and the environmental impacts could be further improved. Using HPC/UHPC leads to a lifetime that is unlikely to be used in real conditions. We assume that the elements will be replaced for aesthetic reasons before the material disintegrates. The environmental impacts could be improved at two other levels: the use of more environmentally friendly materials and the recycling of TRC. Cement and superplasticizer play a major role in the textile concrete facade. An interesting topic to explore would therefore be the partial replacement of cement with other materials, as well as a change in the plasticizer, with a detailed comparison in terms of durability and life cycle assessment.

## 5. Conclusions

The present study aimed to compare the subtle TRC facade elements made of three different types of technical textile rovings (glass, carbon, and basalt) with ordinary facades reinforced by steel reinforcement (ORC) in terms of selected basic environmental impact potentials using an LCA technique that also included a life cycle data inventory. In conclusion, after a detailed calculation and analysis of the whole life cycle, textile reinforced concrete facades appear to be more environmentally friendly in comparison to the ordinary solution in four impact categories by an average of 30%. Ozone depletion (ODP) shows an increase due to the use of plasticizers based on polycarboxylates, which have a great influence on this potential. Carbon has higher results from all compared TRC solutions because of the demanding production process. Carbon fiber production has the greatest effect on abiotic depletion, which is twice as high as that of the ORC solution. The remaining impact categories show very good results for TRC. In general, TRC proves to have very good potential for sustainable construction and environmental impacts for the given conditions and not only for facades. Its use can be applied to similar subtle non-bearing elements. A topic of further research could be its use for load-bearing elements. However, this is subject to further examination and implementation of the relevant standards.

## Figures and Tables

**Table 1:** Description of mixture components of high-performance concrete (HPC) and ordinary concrete (OC) used for textile reinforced facade panels.

HPC		OC	
Component	[kg/m^3^]	Component	[kg/m^3^]
Technical sand	979	Sands	1150
cement I 42.5R	693	Cement CEM II/B-M (S-LL) 32.5	360
quartz flour	332	gravel	810
Silica fume	178	-	-
superplasticizer	29.6	superplasticizer	2.7
water	174	water	155
total	2385.6	total	2477.7

**Table 2:** Technical data of textile reinforcements.

Type of Roving	Linear Density of Roving [Tex]	Tensile Strength [MPa]	Modulus of Elasticity [GPa]	Density [kg/m^3^]	Cross-Sectional Area of Roving [mm^2^]
AR-glass	2400	1700	72	2680	0.896
Carbon	1650	4900	230	1800	0.917
Basalt	2520	2600–2900	85–90	2660	0.947

**Table 3:** Input data for phase 1: Production.

Phase 1 Production Including Assembly						
Input Data		Unit	V1	V2	V3	V4
			ORC	TRC Glass	TRC Carbon	TRC Basalt
Concrete	Concrete ORC (C 30/37)	m^3^	3.600	0	0	0
	Concrete HPC 1	m^3^	0	1.080	1.080	1.080
Concrete components	Cement CEM II/B-M (S-LL) 32.5	t	1.296	0.000	0.000	0.000
	Cement CEM I 42.5 R	t	0.000	0.748	0.748	0.748
	Technical sand	t	0.000	1.057	1.057	1.057
	Sand/gravel	t	7.056	0.000	0.000	0.000
	Silica fume	t	0.000	0.192	0.192	0.192
	Quartz powder	t	0.000	0.359	0.398	0.398
	Super plasticizer (PCE)	t	0.010	0.032	0.032	0.032
	Water	t	0.558	0.188	0.188	0.188
Reinforcement	Steel reinforcement	t	0.266	0.000	0.000	0.000
	Glass reinforcement	t	0.000	0.023	0.000	0.000
	Carbon reinforcement	t	0.000	0.000	0.016	0.000
	Basalt reinforcement	t	0.000	0.000	0.000	0.021
	Epoxy resin treatment	t	0.000	0.014	0.014	0.014
Transport	Transport (long distance > 30 km)	tkm	662	326	324	376
	Transport (short distance < 30 km)	tkm	248	76	76	76

**Table 4:** Input data for phase 2: Use.

Phase 2 Use						
Input Data		Unit	V1	V2	V3	V4
			ORC	TRC Glass	TRC Carbon	TRC Basalt
Concrete	Concrete ORC (C 30/37)	m^3^	0.540	0.000	0.000	0.000
	Concrete HPC 1	m^3^	0.000	0.054	0.054	0.054
Concrete components	Cement CEM II/B-M (S-LL) 32.5	t	0.194	0.000	0.000	0.000
	Cement CEM I 42.5 R	t	0.000	0.037	0.037	0.037
	Technical sand	t	0.000	0.053	0.053	0.053
	Sand/gravel	t	1.058	0.000	0.000	0.000
	Silica fume	t	0.000	0.010	0.010	0.010
	Quartz powder	t	0.000	0.018	0.018	0.018
	Super plasticizer (PCE)	t	0.001	0.002	0.002	0.002
	Water	t	0.084	0.009	0.009	0.009
Reinforcement	Steel reinforcement	t	0.040	0.000	0.000	0.000
	Glass reinforcement	t	0.000	0.001	0.000	0.000
	Carbon reinforcement	t	0.000	0.000	0.001	0.000
	Basalt reinforcement	t	0.000	0.000	0.000	0.001
	Epoxy resin treatment	t	0.000	0.001	0.001	0.001
service	Replacement of facade elements	t	1.378	0.131	0.130	0.131
	Removal	t	1.378	0.131	0.130	0.131
	Water cleaning (ones per 10 years)	t	3.000	3.000	3.000	3.000
Transport	Transport (long distance > 30 km)	tkm	99	16	16	19
	Transport (short distance < 30 km)	tkm	79	8	8	8

**Table 5:** Input data for phase 3: End of life.

Phase 3: End of Life						
Input Data		Unit	V1	V2	V3	V4
			ORC	TRC Glass	TRC Carbon	TRC Basalt
Concrete	Demolition of concrete structure	t	9.2	2.6	2.6	2.6
Transport	Transport (short distance)	tkm	276	78	78	78

**Table 6:** Life cycle inventory analysis data outputs for whole life cycle.

Data Outputs	V1	V2	V3	V4
**Non-Renewable Energy Resources (kg)**	382.1	237.8	401.5	230.0
Crude oil (resource)	110.9	78.6	112.3	80.8
Hard coal (resource)	67.6	38.8	63.1	40.6
Lignite (resource)	70.2	60.0	125.8	54.7
Natural gas (resource)	132.6	60.1	100.1	53.8
Peat (resource)	0.78	0.21	0.22	0.22
Uranium (resource)	0.003	0.001	0.002	0.001
**Non-Renewable Resources (kg)**	15,430	5146	6352	5098
Bauxite	3.76	3.14	2.82	2.73
Bentonite	2.63	1.92	2.02	1.92
Dolomite	3.06	2.17	0.21	0.10
Gypsum (natural gypsum)	45.9	29.08	29.1	29.1
Inert rock	1668	1156	2333	1068
Limestone (calcium carbonate)	1807	1211	1206	1203
Natural aggregate	8970	1315	1317	1316
Natural pumice	52.7	0.001	0.001	0.002
Quartz sand (silica sand; silicon dioxide)	316.9	680.1	709.6	709.9
Sodium chloride (rock salt)	4.02	20.6	22.3	20.6
Soil	2067	578.1	582.4	581.8
**Renewable Resources (kg)**	969,981	566,858	1,049,538	539,285
Water	966,315	564,630	1,044,791	537,151

**Table 7:** Aggregated data for all life cycle on the environmental impacts.

Aggregated DataAll Life Cycle					
Potentials	Unit	V1	V2	V3	V4
		ORC	TRC Glass	TRC Carbon	TRC Basalt
Global warming potential (GWP)	[kg CO_2_ eq.]	1580	1020	1310	1000
Ozone depletion (ODP)	[kg R11 eq.]	0.68 × 10^−6^	2.10 × 10^−6^	2.10 × 10^−6^	2.10 × 10^−6^
Acidification (AP)	[kg SO_2_ eq.]	3.46	2.05	2.35	1.94
Eutrophication (EP)	[kg Phosphate eq.]	0.504	0.329	0.424	0.336
Abiotic depletion (ADP)	[kg Sb eq.]	0.0019	0.0038	0.0018	0.0016
Photochemical oxidant creation (POCP)	[kg Ethene eq.]	0.198	0.141	0.179	0.131

Note: eq. = equivalent.
